# Hare-Lip Operations without Pins

**Published:** 1855-08

**Authors:** 


Hare-Lip Operations without Pins. (Under the care of Mr. Erichsen.)—Cases of hare-lip in general are not very suggestive: the surgeon finds the same kind of deformity in almost every instance, single hare-lip, at the left side, or double hare lip, as the case may be; the same irascible child, tearing the parts open by crying, the same pins and sutures, the same spattering of blood on the surgeon's dress. We are anxious, however, to notice at present one or two points of novelty in this operation, which we have recently noticed in the practice of Mr. Erichsen, at University College Hospital. Chloroform must be used with great caution in all operations about the mouth, lest blood should make its way into the trachea; partial anæsthesia, however, prevents the crying of the child, and if the coronary artery is well caught up in the sutures or pins, there is very little spattering of blood. The chief improvement tried recently in four cases, by Mr. Erichsen, with the most perfect success, is to dispense with the use of pins altogether; the parts are well kept together by ordinary suture, collodion, adhesive plaster, and a particular spring, the invention of a Mr. Hainsbery. The spring, contrived something like a double truss, comes around the head of the child, and presses on the upper maxilla, puckering out the lip and prolabium, and thus prevents the parts being torn open. We have seen this spring also very extensively used by Mr. Ferguson, who has had ninety cases of hare-lip operation with only one failure. Hare-lip pins, we believe, are made of an irritant kind of hard steel, and while transfixing the lip Mr. Erichsen finds they rather act as setons, always leaving ugly marks of suppuration on the lip: the operation being one of what the French call '*convenance*,' or for removing deformity, it cannot be done too nicely. Mr. Erichsen is an advocate for performing the operation early, before the period of dentition.—*London Lancet.*

